# Innovative ICG Application in Benign Gynaecological Surgery: Enhancing Safety and Precision

**DOI:** 10.1155/2024/1642315

**Published:** 2024-07-26

**Authors:** Summer Deah Menezes, Tanushree Rao

**Affiliations:** ^1^ Obstetrics and Gynaecology Department Liverpool Hospital, Locked Bag 7103 Liverpool 2170, Sydney, NSW, Australia; ^2^ University of New South Wales South Western Sydney Clinical School Liverpool Hospital, Level 2, Clinical Building, Locked Bag 7103 Liverpool 2170, Sydney, NSW, Australia

**Keywords:** endometriosis, indocyanine green, laparoscopic surgery

## Abstract

In the context of increased adoption of minimally invasive surgery for benign gynaecological conditions, this study underscores the paramount importance of patient safety. We explored the efficacy of indocyanine green (ICG), a fluorescent dye, in enhancing the visualisation of critical anatomical structures during complex laparoscopic procedures. Our methods involved the direct administration of ICG into the ureters for precise identification and dissection, as well as an innovative vaginal application to delineate the rectovaginal plane in cases with distorted pelvic anatomy. The study presented two cases: a laparoscopic hysterectomy for a multifibroid uterus and a case of advanced endometriosis with rectal involvement. Results indicated that ICG use significantly improved real-time visualisation of the ureters and the rectovaginal plane, which facilitated the surgeries and reduced the cognitive load on surgeons. There were no intraoperative complications, and the postoperative phase showed positive patient outcomes. In conclusion, the application of ICG in these laparoscopic surgeries proved to be a beneficial adjunct, suggesting its potential for broader application in benign gynaecological surgeries. Future research is warranted to explore additional uses of ICG, with a focus on enhancing patient safety and surgical efficacy.

## 1. Introduction

Diagnoses of benign gynaecological conditions have seen a rise in recent years, attributed to heightened awareness and advancements in diagnostic techniques [1]. The preference for minimally invasive surgery in the surgical management of these conditions is growing due to its cosmetic benefits and enhanced postoperative recovery [2–4]. However, the laparoscopic approach's lack of tactile feedback poses a challenge for surgeons in the intraoperative identification and continuous monitoring of critical anatomical structures [4, 5]. This challenge is further amplified in patients with complex pelvic anatomy, such as those with a large multifibroid uterus and late-stage endometriosis, where visibility is obstructed and cognitive load is increased.

A significant challenge in laparoscopic gynaecological surgeries is the intraoperative visualisation and delineation of the ureter. The incidence of ureteral injury in laparoscopic gynaecological surgeries is reported to be between 0.33% and 1.1% [6–9], which escalates to as much as 2.7% in patients with distorted pelvic anatomy, including those with endometriosis and large pelvic masses [8–11]. Such iatrogenic injuries are among the most severe complications of pelvic surgeries, leading to significant morbidity, including sepsis, urinary tract fistulas, varying degrees of renal function impairment, and an increased risk of 90-day hospital readmissions and 1-year mortality [7–9, 11, 12].

Another notable challenge, especially in cases of advanced endometriosis involving the bowel, is delineating the rectum-vagina plane. Major complications of rectal endometriosis surgeries include the formation of rectovaginal fistulas, predominantly due to vaginal injuries, inadequate tissue thickness between the rectum and vagina, or tissue ischemia postsurgery [13–15]. Even when opting for a more conservative rectal shaving approach over disk excision or radical bowel segment resection, the rates of rectovaginal fistulas have been reported to range from 0.25% to 2.1%, with higher rates associated with concurrent vaginal excision [14, 16–20]. Such fistulas significantly deteriorate the quality of life for patients, causing symptoms like flatulence or stool passage through the vagina, recurrent vaginal or genitourinary infections, and dyspareunia [15].

These complications significantly increase the morbidity burden on patients, healthcare professionals, and the healthcare system, thus presenting a considerable clinical challenge in benign gynaecological surgery. Tools that enhance the visualisation of these crucial structures during laparoscopic surgery could lead to improved safety, precision, and optimal patient outcomes.

Indocyanine green (ICG), a water-soluble fluorescent dye, has potential applications in gynaecological laparoscopic surgery for the intraoperative visualisation and navigation of important structures. Initially approved by the FDA in 1959 for clinical use in measuring hepatic blood flow, choroidal blood flow, and cardiac output [21], ICG has been increasingly used in general surgery due to its low cost, favourable side effect profile, and absence of ionising radiation [22]. Its use in gynaecological oncology for sentinel node biopsy is well-established [23, 24], and more recently, the application of ICG in benign gynaecological surgery is being explored.

Near-infrared (NIR) ICG vision has been particularly beneficial in the surgical management of endometriosis. It facilitates the evaluation of residual blood supply after bowel or ureteral surgery, ensuring that tissues maintain adequate perfusion postsurgery [25]. Furthermore, NIR ICG vision aids in the identification of occult lesions that might not be visible under conventional white light, thus improving the thoroughness of endometriosis resection [26, 27]. Additionally, recent advancements include computer quantitative analysis of fluorescent parameters, which provides a more precise and objective assessment of tissue perfusion and viability during surgery [28].

ICG's potential benefits include intraoperative ureter identification through direct administration with cystoscopy and ureteral catheterisation. Several studies have highlighted the advantages of using ICG for real-time visualisation of the ureters, especially in cases with complex pelvic anatomy [21, 29–35]. These studies report enhanced intraoperative safety, precision, and decision-making, leading to improved surgical and patient outcomes, including the elimination of iatrogenic ureteral injuries. Similarly, ICG's use could extend to guiding the delineation of the rectum-vaginal plane in advanced endometriosis cases. This innovative application may improve intraoperative decision-making and reduce rectovaginal fistula rates. To our knowledge, this application has not been previously completed or published.

We present two successful laparoscopic surgeries that utilised ICG in patients with complex benign gynaecological conditions. The first case involved a complex laparoscopic hysterectomy for a large multifibroid uterus, where ICG provided real-time ureter visualisation despite the distorted pelvic anatomy. The second case involved late-stage endometriosis with rectal involvement, where a novel technique of ICG administration through a vaginal spray was used to delineate and maintain the rectum-vaginal plane.

## 2. Case Reports

### 2.1. Case 1: ICG Use in Ureter Identification in a Case of Enlarged Multifibroid Uterus

**Figure 1:**
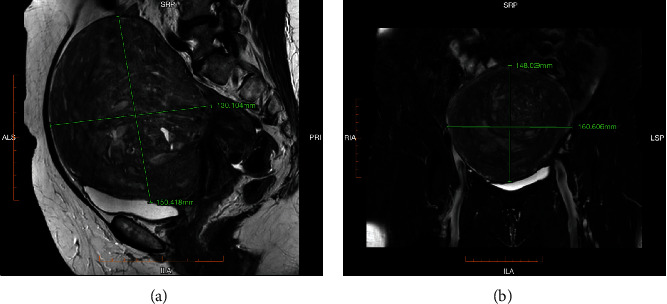
MRI of the pelvis. (a, b) Large leiomyoma arising anteriorly from the body and cervix of the uterus, measuring 150 × 130 × 150 mm, with central cystic and necrotic changes and displacement of the uterus left posteriorly. Multiple smaller fibroids up to 25 × 20 mm and mild distension and dilatation of both ureters also seen.

A 54-year-old woman presented with a history of polyuria, urge and stress incontinence, abnormal uterine bleeding, and pelvic pressure. Her past medical history included a tubular adenoma discovered on colonoscopy, previous left ovarian cyst, and an open appendectomy. Ultrasound imaging of the pelvis revealed a large pedunculated leiomyoma in the posterior aspect of the uterus. Magnetic resonance imaging confirmed a 150 × 130 × 150 mm leiomyoma with central cystic change causing displacement of the uterus left posteriorly and mild distention and dilatation of both ureters (Figure 1).

A total laparoscopic hysterectomy and bilateral salpingectomy were planned, and the patient was commenced on Zoladex in the interim. Due to the enlarged uterus, difficulty in access and visualisation of important structures was anticipated. Hence, the patient consented to the use of ICG for ureter visualisation, administered through cystoscopy and ureteral catheterisation.

**Figure 2:**
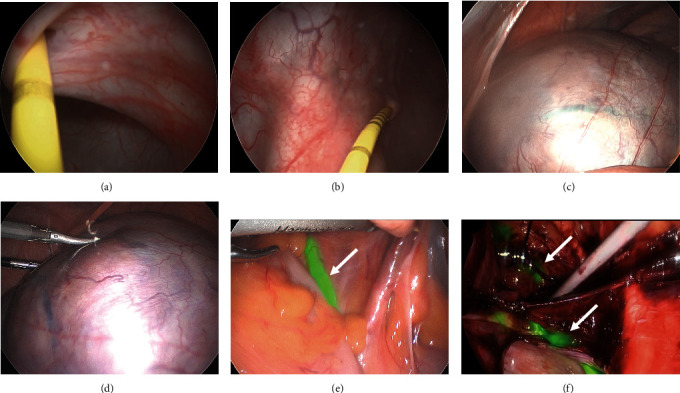
Intraoperative images for Case 1. (a, b) ICG was administered into the ureters bilaterally using cystoscopy and ureteral catheterisation. (c, d) Initial laparoscopic views were obstructed by the large multifibroid uterus filling the pelvic cavity. (e, f) ICG in the ureters illuminated their path on laparoscopic view.

During the surgery, 5 ml of diluted ICG was administered bilaterally into the ureters using cystoscopy and ureteral catheterisation (Figures 2(a) and 2(b)). As anticipated, the initial laparoscopic views were obstructed by a sizable uterus that filled the entire pelvic cavity, complicating the visualisation of crucial anatomical structures (Figures 2(c) and 2(d)). Notably, the large anterior wall fibroid had obliterated the pelvic spaces, particularly the right pararectal and right paravesical spaces, presenting as a pseudo broad ligament fibroid. Nonetheless, the employment of ICG to illuminate the ureters allowed for their safe and efficient dissection (Figures 2(e) and 2(f)). The continuous visual confirmation of the ureters' position via ICG considerably reduced the cognitive burden on the surgeons. This facilitated enhanced precision and safety throughout the procedure, culminating in the completion of the hysterectomy and bilateral salpingectomy in a reduced surgical timeframe and without intraoperative complications.

The patient had no postoperative complications and was discharged the day after surgery. Over 8 months of follow-up, the patient reported complete improvement in urinary symptoms and no further complications.

### 2.2. Case 2: ICG Use in Delineation of the Rectum-Vaginal Plane in a Case of Advanced Endometriosis

A 39-year-old woman was referred to Liverpool Hospital with a 1-year history of dull intermittent left lower quadrant abdominal pain on a background of a known left ovarian endometrioma. The patient's previous menstrual history involved irregular, heavy, and painful periods, associated with dyschezia and deep dyspareunia. Since commencement of Levlen 4 months prior, although her flow had become regular and light, her remaining symptoms showed no improvement. Her past medical and surgical history included chronic hepatitis B managed with entecavir, two lower segment caesarean sections, and an open appendectomy. She also had a family history of chronic hepatitis B, ovarian cancer, and liver cancer. Abdominal and bimanual pelvic examinations found a palpable firm mass in the left lower quadrant which was mildly tender.

**Figure 3:**
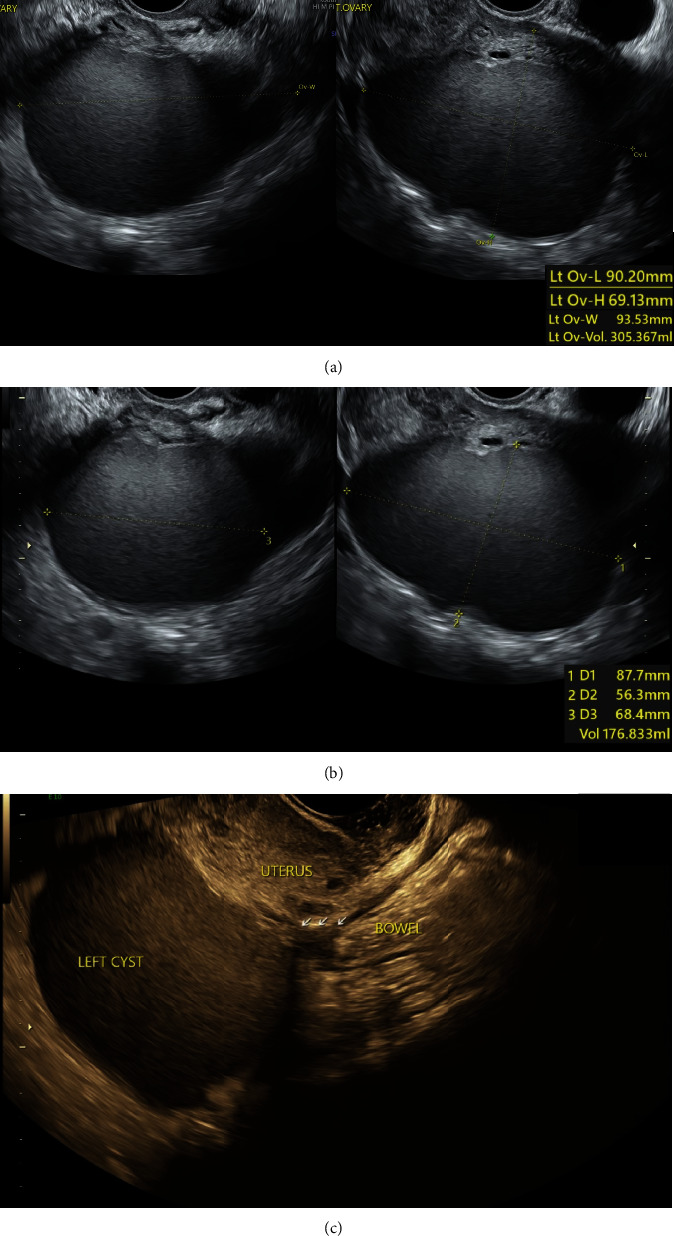
Deep infiltrative endometriosis scans. (a) Enlarged left ovary measuring 90 × 94 × 69 mm with (b) an associated ovarian endometrioma measuring 88 × 68 × 56 mm situated in the Pouch of Douglas. Scans also revealed bowel involvement, with (c) a free-lying loop of bowel tethered to the endometrioma at 160 mm from the anal verge, measuring 10 × 3 × 7 mm.

Pelvic ultrasound scans showed a progressively enlarging left ovarian cyst consistent with an endometrioma, as well as multiple intrauterine fibroids. A deep infiltrative endometriosis scan confirmed an enlarged left ovary at 90 × 94 × 69 mm (Figure 3(a)) with an ovarian endometrioma extending into the Pouch of Douglas measuring 88 × 68 × 56 mm (Figure 3(b)). In addition, the scans revealed bowel involvement, with a free-lying loop of bowel tethered to the endometrioma (Figure 3(c)), as well as concurrent widespread adenomyosis. Thus, advanced Stage 4 endometriosis with bowel involvement was suspected, and the patient was counselled on medical and surgical management options. She consented to a left ovarian cystectomy and bowel adhesiolysis and commenced on Zoladex in the interim until surgery.

However, while awaiting surgery, the patient presented to the emergency department with severe 7/10 left lower quadrant abdominal pain associated with urinary symptoms, including difficulty in initiation and complete voiding. Ultrasound scans revealed further enlargement of the endometrioma to 103 × 93 × 65 mm and an associated mass effect with rightward deviation of the uterus. At this admission, the patient requested a total hysterectomy and bilateral oophorectomy, as she had completed her family and was concerned about continued pain and risks of ovarian cancer. She was counselled extensively on surgical options, particularly in the context of continued dysmenorrhoea due to adenomyosis and risks associated with bilateral oophorectomy at her age. She eventually consented to a total laparoscopic hysterectomy, bilateral salpingectomy, left oophorectomy, and rectal shaving of bowel endometriosis. Due to the patient's advanced endometriosis and multifibroid uterus, a difficult dissection was anticipated. Hence, the use of ICG for ureteral identification and a novel technique of a vaginal spray was suggested and discussed extensively with the patient to which she consented.

**Figure 4:**
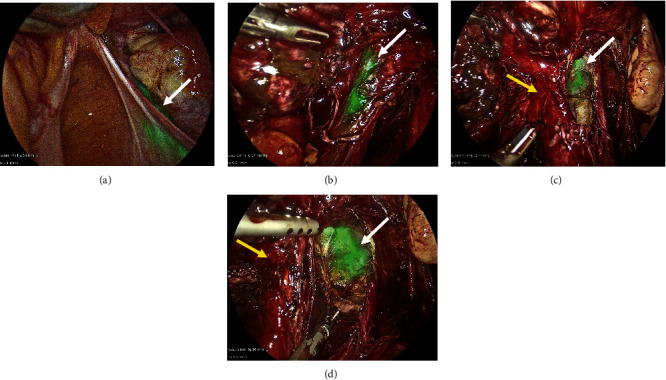
Intraoperative images for Case 2. (a, b) ICG in the ureters illuminated their path on laparoscopic view. (c, d) Vaginal administration of ICG allowed bright fluorescence of the vagina (white arrow), facilitating its delineation from the rectum (yellow arrow) during the surgery.

During the surgery, cystoscopy and ureteral catheterisation were used for the administration of 5 mL of ICG into the ureters bilaterally. A novel technique of a vaginal spray was used to administer 10 mL of ICG into the vagina to facilitate delineation of the rectum-vagina plane. To the best of the authors' knowledge at the time of writing, this is the first time that vaginal administration of ICG for delineation of the rectovaginal border has been conducted or presented. On laparoscopic view, the ureters and vagina were brightly illuminated by ICG, allowing their continued visualisation throughout the surgery (Figure 4). This reduced the cognitive load on the surgeons and enhanced intraoperative decision-making, allowing successful and efficient ureterolysis, left oophorectomy, hysterectomy, and bilateral salpingectomy, with no damage to surrounding structures. In particular, the dissection of the rectal endometrial deposits was facilitated by the fluorescence of ICG in the vagina (Figures 4(c) and 4(d)), which enabled a high level of precision with optimal preservation of healthy tissue. Rectal integrity was tested using the air leak test with no abnormalities detected, and visual examination of the ureters bilaterally identified normal vermiculation with no injuries. All specimens were sent for histological investigation.

The patient's postoperative recovery was uneventful, with only mild pain at surgical sites reported which was controlled with 5 mg of Endone PRN. As she was able to mobilise independently and was keen to return home, the patient was discharged later that day. Her follow-up appointments showed no complications, resolution of her abdominal pain, dyschezia, and dyspareunia, and no further hospital admissions. Histological reports of specimens confirmed focal uterine serosa endometriosis, left ovarian endometriotic cyst, and rectal endometriosis.

## 3. Discussion

In recent years, there has been a burgeoning demand for minimally invasive surgery in benign gynaecological cases. Nevertheless, this laparoscopic approach presents significant challenges, particularly in identifying and preserving vital anatomical structures. These difficulties are exacerbated in instances of distorted pelvic anatomy and extensive disease, which obscure visibility and add complexity to the surgical procedures. ICG, a fluorescent dye with established clinical applications, has begun to be utilised in benign gynaecological surgeries to potentially enhance intraoperative visualisation of key structures, thereby improving precision and patient safety. In this context, we have presented two intricate benign gynaecological cases where ICG facilitated the real-time visualisation of essential anatomical landmarks.

The administration of ICG directly into the ureters enabled safer and more accurate identification and dissection of these structures in both patients, despite their complex and distorted pelvic anatomy. Integrating this technology markedly reduced the cognitive load on surgeons, delivering enhanced visual clarity that fostered confident navigation through these intricate pelvic surgeries, thus improving both surgical and patient outcomes. The initial use of ICG for ureteral localisation during robot-assisted ureteroureterostomy was reported by Lee et al. [29], where it demonstrated its efficacy in distinguishing healthy tissue from diseased tissue. In their pilot study, all patients experienced clinical resolution without any ICG-related complications over a 6-month follow-up. Successive reports by Siddighi, Yune, and Hardesty and Park and Farnam echoed these findings in robotic-assisted laparoscopic surgeries, with no intraoperative or postoperative complications reported [30, 31]. Subsequent small cohort studies and case reports have consistently confirmed the efficacy of ICG in patients with complex pelvic anatomy, achieving a 100% success rate in ureteral identification even when traditional white light visualisation was insufficient [21, 32–35]. Furthermore, these studies noted improved surgical metrics and patient outcomes with the use of ICG, such as minimised blood loss due to reduced unnecessary dissection, shorter surgical duration, resolution of preoperative symptoms, and an absence of complications [21, 32–35]. Our study's findings are in harmony with this accumulating evidence on the effectiveness of ICG for ureteral identification. Additionally, previous experiences with fluorescence urography for endometriosis provide valuable insights and differences that further emphasize the innovative application of ICG in our cases [36].

While alternative techniques for intraoperative ureter identification, such as conventional double J ureteral stent placement and methylene blue (MB) injection, have been proposed, recent studies indicate a lack of clinical benefit of preoperative stenting on postoperative complications [37]. Double J stents can be inserted preoperatively for palpation or visualisation during surgery. However, they are associated with increased complications and are less practical for laparoscopic surgery due to the absence of tactile feedback [32, 38, 39]. MB and ICG are the only fluorophores currently approved by the FDA for clinical use. Consequently, studies have compared their efficacy [38, 40–42]. MB can be administered intravenously and is excreted renally, which, unlike ICG that requires direct ureteral catheterization, may seem advantageous. Nevertheless, intravenous MB has a delayed detection time in the ureters and is less effective in patients with compromised renal function. Moreover, MB is subject to more nonspecific background fluorescence and has a lower tissue perfusion capacity compared to ICG [40, 43]. Overall, these alternative methods are relatively more costly and time-consuming than ICG and do not provide the same level of high-quality real-time ureteral visualisation [21, 32, 33].

In our study, we also report a novel application of ICG delivered through a vaginal spray to delineate the rectum-vaginal border in a complex case of advanced-stage endometriosis with rectal involvement. The vaginal ICG administration illuminated the rectum-vaginal plane, aiding the dissection of rectal endometrial deposits—one of the most challenging aspects of the surgery. This approach enabled surgeons to dissect with greater precision, avoiding inadvertent damage to the vagina or other intraoperative complications. The case concluded successfully, with the patient reporting a smooth recovery, resolution of symptoms, and no postoperative complications. To our knowledge, this technique has not been previously documented. This innovative approach may offer additional benefits in gynaecological surgeries, particularly in scenarios like advanced endometriosis, where distinguishing healthy tissue is notoriously difficult.

## 4. Conclusion

As the shift towards more minimally invasive surgeries for benign gynaecological conditions continues, the imperative of patient safety remains at the forefront. In complex gynaecological cases, surgeons face a cognitive overload due to the risk of injury to adjacent structures. ICG proves to be an invaluable aid in enhancing the visibility of critical structures, thereby facilitating improved precision and safety. This study showcased two intricate laparoscopic cases with distorted pelvic anatomy where the use of ICG enabled clear and real-time visualisation of vital healthy structures, including a pioneering technique of vaginal ICG administration for the delineation of the rectovaginal plane. This innovation holds promise not only for patient benefit but also as a significant contribution to the ongoing advancement of gynaecological surgical techniques. Future research should explore additional applications of ICG, aimed at further refining our minimally invasive surgical skills to heighten patient safety and optimise health outcomes.

## Data Availability

The clinical data used to support the findings of this study are included in this article.

## Supporting Information

Supporting Information Additional supporting information can be found online in the Supporting Information section. Supporting video: presentation of case studies accompanied by surgical videos. https://drive.google.com/file/d/1eQX80mLeuIRfmyoXX-e7cSGm7Jtf_vm2/view?usp=sharing.
