# Supplementary material for: Innovative ICG Application in Benign Gynaecological Surgery: Enhancing Safety and Precision
Source: Case Rep Obstet Gynecol. 2024 Jul 26;2024:1642315. doi: 10.1155/2024/1642315 (PMC11300066; doi:10.1155/2024/1642315)
Supplement: Supporting Information — Additional supporting information can be found online in the Supporting Information section. Supporting video: presentation of case studies accompanied by surgical videos. https://drive.google.com/file/d/1eQX80mLeuIRfmyoXX-e7cSGm7Jtf_vm2/view?usp=sharing. [file 1642315.f1.pdf]

Supplementary Video: presentation of case studies accompanied by surgical videos, including novel surgical technique of the use of indocyanine green through a vaginal spray for delineation of the rectum-vaginal plane.

https://drive.google.com/file/d/1eQX80mLeuIRfmyoXX-e7cSGm7Jtf_vm2/view?usp=sharing
